# Supplementary material for: Asynchronous magnetic resonance elastography: Shear wave speed reconstruction using noise correlation of incoherent waves
Source: Magn Reson Med. 2022 Oct 27;89(3):990–1001. doi: 10.1002/mrm.29502 (PMC9792433; doi:10.1002/mrm.29502)
Supplement: Supplementary file 1 — DATA S1 MATLAB code used to generate the numerical simulations of Figure 1 [file MRM-89-990-s002.zip › k-Wave/helpfiles/example_ivp_comparison_modelling_functions.html]

Comparison Of Modelling Functions Example (k-Wave)


# Comparison Of Modelling Functions Example

This example provides a short comparison between the simulation functions `kspaceFirstOrder2D` and `kspaceSecondOrder`. It builds on the Homogeneous Propagation Medium and Using A Binary Sensor Mask examples.

- Open the file in the MATLAB Editor
- Run the file in MATLAB

For a more detailed discussion of the second order model used in k-Wave, see Treeby, B. E. and Cox, B. T., "A k-space Green's function solution for acoustic initial value problems in homogeneous media with power law absorption," *J. Acoust. Soc. Am.*, vol. 129, no. 6, pp. 3652-3660, 2011.

## Contents

- About the modelling functions
- Running the simulation
- Numerical comparison

## About the modelling functions

In the previous examples, the acoustic simulations are all performed using `kspaceFirstOrder2D`. This function is based on the sequential calculation of the particle velocity, acoustic density, and acoustic pressure using three coupled first-order partial differential equations (conservation of mass, conservation of momentum, and a pressure-density relation). For a homogeneous medium, these equations can also be combined into a single second-order acoustic wave equation and solved using Green's function methods. The function `kspaceSecondOrder` is an efficient numerical implementation of a Green's function solution specifically for initial value problems. For homogeneous media, the two approaches give the same results.

Compared to `kspaceFirstOrder2D`, the function `kspaceSecondOrder` has greater computational efficiency and the time steps can be arbitrarily large (as the solution is exact). It also allows both an initial pressure and an initial pressure gradient to be defined (see the Setting An Initial Pressure Gradient Example). Power law absorption is also more accurately encoded (see the Modelling Power Law Absorption Example). However, `kspaceSecondOrder` has slightly less functionality than its first-order counterparts (`kspaceFirstOrder1D`, `kspaceFirstOrder2D`, and `kspaceFirstOrder3D`). For example, it only supports homogeneous media and binary sensor masks, and does not support time varying sources or the full range of visualisation options.

The second-order code does not implement an absorbing boundary layer (see Controlling The Absorbing Boundary Layer Example for more details on the perfectly matched layer used in `kspaceFirstOrder2D`). Instead, to delay the appearance of wave wrapping, it extends the size of the computational grid. Although this reduces the computational efficiency, it allows the exactness of the solution to be maintained. The grid extension is controlled by setting the optional input parameter `'ExpandGrid'` to `true`. Note, the wrapped waves will still appear if the simulation time is longer than the time it takes for the waves to propagate through the grid extension.

## Running the simulation

The function `kspaceSecondOrder` (this is used for all dimensions) takes the same input structures as the first-order simulation functions. For example, to run the Homogeneous Propagation Medium Example, the Cartesian sensor mask is first converted to a binary sensor mask using `cart2grid`, and then `kspaceSecondOrder` is called with `'ExpandGrid'` set to `true`.

```
% convert the cartesian sensor mask to a binary sensor mask
sensor.mask = cart2grid(kgrid, sensor.mask);

% run the simulation using the second order code
sensor_data = kspaceSecondOrder(kgrid, medium, source, sensor, 'ExpandGrid', true);
```

## Numerical comparison

When the medium is not absorbing and no compensation for wave wrapping is used, the two simulation functions give identical results to machine precision. A plot of the signals recorded at the first sensor position and the difference between them is given below (set `example_number = 1` within the example m-file).

When the medium is absorbing and wave wrapping is mitigated using a perfectly matched layer, there is a small error in the numerical result computed using `kspaceFirstOrder2D`. This error is largely due to the calculation of the power law absorption term (see the Modelling Power Law Absorption Example). A plot of the signals recorded at the first sensor position and the difference between them is given below (set `example_number = 4` within the example m-file).
